# Estimation of reinforced urn processes under left-truncation and right-censoring

**DOI:** 10.1098/rsos.221223

**Published:** 2023-03-08

**Authors:** Luis A. Souto Arias, Pasquale Cirillo, Cornelis W. Oosterlee

**Affiliations:** ^1^ Mathematical Institute, Utrecht University, Utrecht, The Netherlands; ^2^ ZHAW School of Law and Management, Zurich University of Applied Sciences, Zurich, Switzerland

**Keywords:** reinforced urn process, expectation–maximization, bivariate survival function, left-truncation, right-censoring

## Abstract

We propose a non-parametric estimator for bivariate left-truncated and right-censored observations that combines the expectation–maximization algorithm and the reinforced urn process. The resulting expectation-reinforcement algorithm allows for the inclusion of experts’ knowledge in the form of a prior distribution, thus belonging to the class of Bayesian models. This can be relevant in applications where the data is incomplete, due to biases in the sampling process, as in the case of left-truncation and right-censoring. With this new approach, the distribution of the truncation variables is also recovered, granting further insight into those biases, and playing an important role in applications like prevalent cohort studies. The estimators are tested numerically using artificial and empirical datasets, and compared with other methodologies such as copula models and the Kaplan–Meier estimator.

## 1. Introduction

We deal with bivariate left-truncated and right-censored (LTRC) data in a non-parametric way, combining reinforced urn processes (RUP) [1]—in particular, the bivariate construction (B-RUP) of Bulla *et al.* [2]—and the expectation–maximization (EM) algorithm. We call this combination the expectation-reinforcement (ER) algorithm, which enjoys the optimization properties of the EM algorithm, while maintaining the Bayesian formulation of the RUP. The ER approach proves preferable over the classic EM when reliable experts’ knowledge about the phenomenon under study is available, which can then be included as a prior distribution. This feature enables the correction of biases present in the data, especially when dealing with extreme and rare events, as well as the possibility of inputting plausible information about future trends and other not-yet-observed characteristics.

LTRC observations are frequent and relevant in several fields of science, such as medicine, survival studies, education studies, engineering and risk management [3–5]. For instance, right-censoring appears in most medical studies, as patients are observed over a limited period of time, and the event of interest—e.g. the next stage of a disease—may occur after the observation period. Left-truncation, conversely, occurs when patients join the study with the disease already in an advanced stage, giving rise to missed information about the earlier stages [6].

The problem of non-parametric estimation in the bivariate LTRC setting is not new, and among the first proposals to tackle this problem one can find [7–9] and [10]. However, as shown in [11], these estimators may fail to be monotone, thus possibly generating negative probabilities. More recent works are those of Shen & Yan [5] and Gribkova & Lopez [12]. The former develops a complex iterative method to estimate a generalization of the Dabrowska and Campbell and Földes estimators, which includes the effect of left-truncation. A simplified version of this algorithm that does not require iteration was proposed in [13]. In spite of this numerical advantage, the new estimator developed in [13] can be sensitive with respect to some of its parameters, producing inaccurate results when not chosen properly. Gribkova & Lopez [12] use a non-parametric estimator via random weights, first defined in [14], to compute a non-parametric copula in the presence of bivariate right-censoring when there is no left-truncation. The case of bivariate left-truncation is particularly interesting, since, as explained in [5], in this case the Kaplan–Meier (KM) and product-limit estimators [15,16] are not consistent estimators of the marginal survival functions. Specifically, in the bivariate setting with two target variables (*X*, *Y*), as shown in [5], the product-limit estimator computes the marginal distribution of *X* conditioned on the truncation event of *Y*, which does not correspond to the true marginal distribution of *X*. A similar reasoning applies to the estimation of the marginal distribution of *Y*. Therefore, not only the joint distribution, but also the marginal distributions are difficult to determine in the presence of multivariate left-truncation.

Since left-truncation and right-censoring fall under the umbrella of incomplete data, many authors have opted for an alternative approach, using the EM algorithm of Dempster *et al.* [17]. Some relevant results in the univariate framework are available in [4] and references therein. Conversely, the literature on bivariate distributions is scarce, focusing mainly on right-censoring (see [9,18,19]). In fact, to our knowledge, there are no proposals in the literature of the EM algorithm that include bivariate left-truncation.

It should be noted that the authors in [20] have already shown that the B-RUP is able to model bivariate censoring in practical applications, comparing its performances to parametric approaches based on copulas [21,22]. However, the approach followed in [20], based on Markov Chain Monte Carlo (MCMC) simulation, is limited to the modelling of right-censoring, omitting the effect of left-truncation.

In fact, in many applications, relevant information can be extracted from the distribution of the truncation variables (see [23]). This is the case for prevalent cohort studies where, under certain assumptions, the truncation distribution corresponds to the disease distribution prior to recruitment. The proportion of truncated observations is another quantity of interest. In medical studies, for example, this quantity corresponds to the number of individuals that have died due to a specific disease prior to recruitment.

Our contribution contains, therefore, three main novelties, which can be summarized as follows:

— We offer an effective treatment of non-parametric distributions under bivariate LTRC data, overcoming some difficulties of the existing literature. The approach we propose for LTRC data can be easily adapted to the simpler situations of just LT or RC data, and to distributions in higher dimensions with minor adaptations in the methodology.
— We present an approach for the explicit estimation of RUPs in particular, and urn models in general. In the literature, urn models [24,25] have been mainly approached from a probabilistic point of view, and statistical inference has always been marginal, with some exceptions such as [26–28].
— To fully exploit the Bayesian properties of RUPs [29], we introduce the ER algorithm, which maintains the convergence properties of the standard EM algorithm but also enables the inclusion of experts’ knowledge.

The paper is structured as follows. In §2, we briefly revisit the concepts of right-censoring and left-truncation and introduce the modelling assumptions. Section 3 summarizes the basic theory of RUPs, as well as the bivariate one-factor construction (B-RUP) of Bulla *et al.* [2], and their adaptation to LTRC data. The EM and the ER algorithms for RUPs are described in §4, and complemented by an error analysis. Section 5 contains the performances of the algorithms using both simulated and empirical data. Finally, §6 concludes the paper.

## 2. Left-truncation and right-censoring

Let ***X***_*n*_ = (*x*_1_, …, *x*_*n*_) and ***C***_*n*_ = (*c*_1_, …, *c*_*n*_) be identically and independently distributed (i.i.d) observations with distribution functions *F*_*X*_ and *F*_*C*_, respectively. *F*_*X*_ and *F*_*C*_ are assumed independent.

If right-censoring occurs, we observe the pair $\left(x_{i}^{*},\delta_{i}\right)$, where $x_{i}^{*}=\min(x_{i},c_{i})$ and $\delta_{i}=1_{\left\{x_{i}^{*}=x_{i}\right\}}$ for *i* = 1, …, *n*, with $1_{\left\{\cdot \right\}}$ the indicator function. That is, we observe the minimum of the censoring variable and the target variable, plus an indicator telling us which of the two we observe.

Let ***T***_*n*_ = (*t*_1_, …, *t*_*n*_) be another i.i.d. sequence with distribution *F*_*T*_, independent of *F*_*X*_. When left-truncation occurs, we observe the pair (*x*_*i*_, *t*_*i*_), for *i* = 1, …, *n*, if *x*_*i*_ ≥ *t*_*i*_, and nothing otherwise.

Since for *x*_*i*_ < *t*_*i*_ nothing is observed, the data contains no information about *X* or *T* for *X* < *T*. This suggests that a truncated observation provides less information than a censored one, since for cases where P(T≤X) is small, the truncated sample will be highly biased with respect to the original underlying distribution. For this reason, according to Wang [30], truncated data can also be classified as selection-biased data.

In the general case of LTRC observations, it is usually assumed [30,31] that the variable of interest *X* is independent of both *T* and *C*. In this situation what one observes is the triplet $\left(X_{n}^{*},T_{n},\delta_{n}\right)$, with X* and ***δ*** defined as before if *T* ≤ *X*, and nothing otherwise. As in [30], we further assume that P(T≤C)=1, indicating that *T* and *C* are not independent.^1^ In this case, the log-likelihood of the sample can be written as

2.1 $$L(X_{n}^{*}|\delta_{n},T_{n})=\sum_{i=1}^{n}[(1-\delta_{i})\log(P(X>x_{i}^{*}))+\delta_{i}\log(P(X=x_{i}^{*}))-\log(P(X\geq t_{i}))].$$

In the case of non-negative integers, a non-parametric estimator for the survival function, *S*_*X*_, which maximizes the log-likelihood defined in equation (2.1) was introduced in [31] and further studied in [16], is given by

2.2 $$S_{X}\;:=P(X>x|X_{n}^{*},T_{n},\delta_{n})=\prod_{\;j=0}^{x}\left[1-\frac{m_{j}(X_{n}^{*},\delta_{n})}{s_{j}(X_{n}^{*},T_{n})}\right],$$

where $m_{j}(x_{n},d_{n})=\sum_{i=1}^{n}1_{\left\{x_{i}=j,d_{i}=1\right\}}$ is the number of exact observations at *j*, and $s_{j}(x_{n},t_{n})=\sum_{i=1}^{n}1_{\left\{t_{i}\leq j\leq x_{i}\right\}}$ is the number of censored plus uncensored observations at *j* under left-truncation. Equation (2.1) reduces to the well-known KM estimator in the absence of truncation [15], and to the product-limit estimator of Lynden-Bell [32] without censoring.

**Remark.**

In this article, we work with non-negative integers unless otherwise stated. This is done for simplicity, and the extension from the non-negative integers to a discrete set of the real line is straightforward.

In the bivariate situation, we assume that the joint survival function *S*_*XY*_ of the variables (*X*, *Y*) is independent of the truncation and censoring variables (*T*^*X*^, *C*^*X*^, *T*^*Y*^, *C*^*Y*^) (see [5]). Observations consist of two triplets $\left(X_{n}^{*},T_{n}^{X},\delta_{n}^{X}\right)$ and $\left(Y_{n}^{*},T_{n}^{Y},\delta_{n}^{Y}\right)$. If the target variables *X* and *Y* are independent, we can write the joint conditional log-likelihood as the sum of the marginal log-likelihoods, both defined as in equation (2.1). However, when there exists dependence, the log-likelihood of the bivariate LTRC sample takes the form:

2.3 $$\begin{array}{rl} & L(X_{n},Y_{n}|{\delta^{X}}_{n},{\delta^{Y}}_{n},T_{n}^{X},T_{n}^{Y})=\sum_{i=1}^{n}[\log(P^{*}(x_{i}^{*},y_{i}^{*}|\delta_{i}^{X},\delta_{i}^{Y}))-\log(P(X\geq t_{i}^{X},Y\geq t_{i}^{Y}))], \end{array}$$

with

2.4 $$P^{*}(x,y|\delta^{X},\delta^{Y})=\left\{ \begin{array}{cc} P(X=x,Y=y) & \text{if }\delta^{X}=1\text{ and }\delta^{Y}=1,\\ P(X>x,Y=y) & \text{if }\delta^{X}=0\text{ and }\delta^{Y}=1,\\ P(X=x,Y>y) & \text{if }\delta^{X}=1\text{ and }\delta^{Y}=0,\\ P(X>x,Y>y) & \text{if }\delta^{X}=0\text{ and }\delta^{Y}=0. \end{array}\right.$$

### 2.1. Modelling assumptions

As mentioned in [23], it is not clear how to estimate the joint distribution *H*_*T*,*C*_ of the censoring and truncation variables (*T*, *C*), in the case of LTRC observations, which is, however, necessary for the algorithms developed in §4. For that purpose, Wang [23] makes the assumption, in the univariate setting, that *C* = *T* + Δ, where *T* and Δ are independent random variables (r.v). Under this assumption, the distribution *H*_*T*,*C*_ can be expressed as

2.5 $$dH_{T,C}(t,c)=dH_{T}(t)\;dH_{\Delta }(c-t),$$

where *H*_*T*_( · ) and *H*_Δ_( · ) are the probability distributions of *T* and Δ, respectively.

**Remark.**

Note that the censoring assumption *C* = *T* + Δ implies that censoring is at the end of the follow up, either because the study has finished or because the participant dropped out of the study. This is the case in many practical situations and, in particular, it is true for the annuity problem studied in [20,21], which we treat in §5.2.

In the bivariate LTRC setting, we are interested in the joint distribution *H* of two censoring (*C*^*X*^, *C*^*Y*^) and two truncation (*T*^*X*^, *T*^*Y*^) variables. Therefore, the censoring assumption becomes *C*^*i*^ = *T*^*i*^ + Δ, for *i* ∈ {*X*, *Y*}. Observe that we take the same Δ for *T*^*X*^ and *T*^*Y*^, thus entailing that both participants end the follow up at the same time. This is the case when the end of the follow up matches the end of the study. On the other hand, it could happen that the two participants drop out at different times during the study. In that case the source of dependence between participants is lost, diminishing the relevance of that particular observation. Another possibility, that we do not pursue in this work, is to set *C*^*X*^ = Δ + *T*^*X*^ + *C*^*Y*^ (see [5,13]). In this case, assuming Δ and *T*^*X*^ non-negative, it is clear that $P[C^{Y}\leq C^{X}]=1$, which can be relevant in scenarios where we are sure that one participant will drop out earlier than the other one. The methodology proposed in §4 can also be applied under this assumption, with minor modifications.

Finally, we make the truncation assumption $T^{Y}=T^{X}+\epsilon$, where *T*^*X*^ and ϵ are independent random variables. This situation arises for example in survival studies, where *T*^*X*^ and *T*^*Y*^ denote the age at which each individual enters the study [20,21]. In this case, the r.v. ϵ corresponds to the age difference between the individuals. Another example where this assumption is verified is in paediatric AIDS cohort studies (see [13]), where ϵ denotes the difference between the time of infection of the mother and the time of birth of the child.

## 3. Univariate and bivariate reinforced urn processes

The RUP and B-RUP models define random processes in one and two dimensions, respectively. In particular, we use the B-RUP to model bivariate LTRC data in a non-parametric way. Since the B-RUP is composed of several RUPs, a brief explanation of the univariate RUP model is also needed to keep this article self-contained. The reader is referred to Walker & Muliere [1], Bulla *et al.* [2], Arias & Cirillo [20] and Muliere *et al.* [33] and the references therein for an extended and more thorough analysis of the theoretical properties of these models.

### 3.1. The reinforced urn process

An interesting property of the RUP is that it can be easily visualized as a series of Pólya urns with balls of two different colours. Thus, assume we have *M* + 1 Pólya urns [25], where the *j*th urn *U*_*j*_, *j* = 0, 1, …, *M*, initially contains *ω*_*j*_ > 0 green (G) balls and *β*_*j*_ > 0 red (R) balls. The only exception is urn *U*_0_, which only has green balls. The number of balls does not need to be an integer.

(i) The process starts in *U*_0_.
(ii) For *j* = 0, 1, …, *M*, randomly pick a ball from urn *U*_*j*_, look at its colour, put the ball back into the urn, and add *r* > 0 balls of the same colour.
(iii) If the colour of the chosen ball is green, move forward to urn *U*_*j*+1_, and repeat from step 2. If the colour is red, go back to step 1.

**Figure 1.**
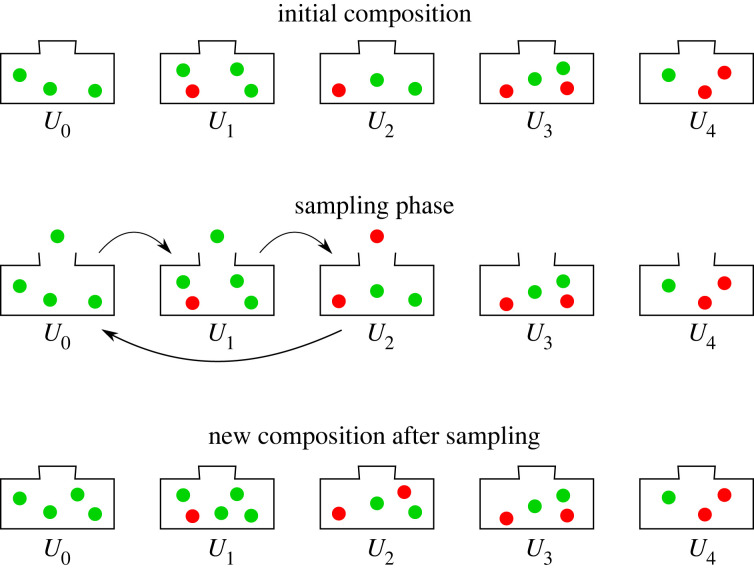
Representation of the RUP as a series of Pólya urns. The balls outside of the urns indicate which colour has been sampled. After each sampling, the urns are updated in a way that reinforces the probability of observing the same event again.

As an example, in figure 1 we see the result of picking (green G ball, green G ball, red R ball) and the resulting urn compositions, if we assume *r* = 1. In many applications, the event of interest is usually the sampling of a red ball. In the context of survival studies, this corresponds to the event of death, while the urn at which this happens corresponds to the lifetime of an object or an individual (if each urn denotes a different year, the observed lifetime in figure 1 would be 2 years). Hence, by observing different lifetimes and reinforcing the urns accordingly, the RUP is able learn from the data and produce an accurate predictive distribution of the event of interest.

Higher values of *r* imply a stronger reinforcement, i.e. the composition of the RUP quickly changes via sampling, while for values of *r* close to zero the RUP requires many iterations in order to move away from its initial composition. The reader is referred to Cirillo *et al.* [34] and Peluso *et al.* [35] for a discussion about the role of *r*.

If the data include right-censoring and left-truncation, the procedure we have just described can be easily modified. In case of a right-censored observation, the red ball is not included, but everything else is left unchanged. If the sample is left-truncated at value *j*, the new cycle starts from urn *U*_*j*_, and not from *U*_0_.

One of the most interesting features of a RUP is that the probability of its trajectories can be obtained in closed form Muliere *et al.* [33] and Fortini & Petrone [36]. This comes from the fact that, as shown in [33], a RUP generates a random distribution over the space of discrete distributions, and this random distribution is the discrete beta-Stacy process of Walker & Muliere [1].

**Definition 3.1 Beta-Stacy process (discrete case).**

A random distribution function *F* is a discrete beta-Stacy process with jumps at $j\in N_{0}$ and parameters ${\left\{\beta_{j},\omega_{j}\right\}}_{\;j\in N_{0}}$, if there exist mutually independent random variables ${\left\{V_{j}\right\}}_{\;j\in N_{0}}$, each beta distributed with parameters (*β*_*j*_, *ω*_*j*_), such that the random mass assigned by *F* to {*j*}, written *F*({*j*}), is given by *V*_*j*_∏_*i*<*j*_ (1 − *V*_*i*_).

Following Walker & Muliere [1], we introduce couples $\{\beta_{j},\omega_{j}\}\in R^{+}\times R^{+}$, with $j\in N_{0}$, such that

— *β*_*j*_, *ω*_*j*_ ≥ 0,
— *β*_*j*_ + *ω*_*j*_ > 0,
— and $\lim_{n\to \infty }\prod_{\;j=0}^{n}(\omega_{j}/\left(\beta_{j}+\omega_{j}\right))=0$.

Then, given a beta-Stacy process *F* with parameters ${\left\{\beta_{j},\omega_{j}\right\}}_{\;j\in N_{0}}$, and an LTRC sample $\left(X_{n}^{*},T_{n},\delta_{n}\right)$, the posterior distribution of the r.v. *X* given by an RUP with reinforcement *r* reads

3.1 $$\hat{S}(x)\;:=P(X>x|X_{n}^{*},T_{n},\delta_{n})=\prod_{\;j=0}^{x}\left[1-\frac{\beta_{j}+r\cdot m_{j}(X_{n}^{*},\delta_{n})}{\beta_{j}+\omega_{j}+r\cdot s_{j}(X_{n}^{*},T_{n})}\right],$$

where *m*_*j*_(***x***_*n*_, ***d***_*n*_) and *s*_*j*_(***x***_*n*_, ***t***_*n*_) are defined as in equation (2.2).

Defining $\beta_{j}^{*}=\beta_{j}+r\cdot m_{j}^{*}(x_{n},d_{n})$ and $\omega_{j}^{*}=\omega_{j}+r\cdot (s_{j}(x_{n},t_{n})-m_{j}^{*}(x_{n},d_{n}))$, we obtain a new beta-Stacy process F* with parameters ${\left\{\beta_{j}^{*},\omega_{j}^{*}\right\}}_{\;j\in N_{0}}$, which implies that the beta-Stacy process—and hence also the RUP—is conjugate to LTRC data. This is proven formally in the following proposition, in which we extend the results of Muliere *et al.* [33] and Walker & Muliere [1], where the authors only focused on right-censored observations.

**Proposition 3.2.**

*The RUP as defined in equation* (*3.1*) *is conjugate to LTRC data*.

**Proof.**

We start by using the fact that the underlying distribution of the RUP is the generalized Dirichlet distribution of Connor & Mosimann [37], i.e.

3.2 $$\begin{array}{rl} & P(p_{X}(0),p_{X}(1),\ldots ,p_{X}(x_{M})|\beta ,\omega )\propto {\left(p_{X}(x_{M})\right)}^{\omega_{x_{M}-1}-1}\times \prod_{\;j=0}^{x_{M}-1}[{\left(p_{X}(j)\right)}^{\beta_{j}-1}{\left(S_{X}(j-1)\right)}^{\omega_{\;j-1}-(\beta_{j}+\omega_{j})}], \end{array}$$

where *p*_*X*_( · ) and *S*_*X*_( · ) denote the probability mass function (PMF) and survival function of *X*, respectively, and *x*_*M*_ is the maximum value *X* can take.

Under this distribution, the likelihood of the LTRC sample $\left(X_{n}^{*},T_{n},\delta_{n}\right)$ is given by

3.3 $$L(X_{n}^{*}|T_{n},\delta_{n})=\prod_{\;j=0}^{x_{M}}{\left(p_{X}(j)\right)}^{m_{j}(X_{n}^{*},\delta_{n})}\prod_{\;j=0}^{x_{M}-1}{\left(S_{X}(j)\right)}^{r_{j}(X_{n}^{*},\delta_{n})-l_{j}(T_{n})},$$

where $r_{j}(x_{n},d_{n})=\sum_{i=1}^{n}1_{\left\{x_{i}=j,d_{i}=0\right\}}$ is the number of censored observations at *j*, and $l_{j}(t_{n})=\sum_{i=1}^{n}1_{\left\{t_{i}\leq j\right\}}$ is the number of truncated observations at *j*.

Since the posterior distribution is proportional to the product of equations (3.2) and (3.3), i.e. the product of the prior distribution and the likelihood, it follows immediately that if *F* is a discrete beta-Stacy process with parameters ${\left\{\beta_{j},\omega_{j}\right\}}_{\;j\in N_{0}}$, by setting $q_{j}=\sum_{i=j+1}^{x_{M}}m_{i}(X_{n}^{*},\delta_{n})+\sum_{i=j}^{x_{M}}r_{i}(X_{n}^{*},\delta_{n})-\sum_{i=1}^{j}l_{i}(T_{n})$, the posterior distribution of *F* given the LTRC data $\left(X_{n}^{*},T_{n},\delta_{n}\right)$ is also a discrete beta-Stacy process with parameters

3.4 $$\beta_{j}^{*}=\beta_{j}+m_{j}(X_{n}^{*},\delta_{n})\;\mathrm{and}\;\omega_{j}^{*}=\omega_{j}+q_{j},$$

and since $q_{j}=s_{j}(X_{n}^{*},T_{n})-m_{j}(X_{n}^{*},\delta_{n})$, this shows that the RUP is conjugate to LTRC data. ▪

Comparing with the urn representation of figure 1, $\hat{S}(x)$ in equation (3.1) corresponds to the probability of selecting at least *x* consecutive green balls, starting from urn *U*_0_. For *j* = 0, 1, 2, …, the couple {*β*_*j*_, *ω*_*j*_} determines the initial number of red and green balls in urn *U*_*j*_, respectively, while the functions $m_{j}^{*}(x_{n},d_{n})$ and *s*_*j*_(***x***_*n*_, ***t***_*n*_) determine the extra number of green and ‘green plus red’ balls added to each urn over time, respectively.

Being a random distribution, an important characteristic of the (discrete) beta-Stacy process is that its trajectories can be centred around a certain (discrete) probability distribution *F*_0_( · ), which plays the role of the prior distribution. As proven in [1], a necessary condition for this to hold is that

3.5 $$\frac{\beta_{j}}{\beta_{j}+\omega_{j}}=\frac{F_{0}(j)-F_{0}(j-1)}{1-F_{0}(j-1)},\;j\in N,$$

where $F_{0}(j)=P_{F_{0}}(X\leq j)$ is the probability that *X* is at most *j* under the prior *F*_0_.

Note that, according to equation (3.5), the choice for the couples ${\left\{\beta_{j},\omega_{j}\right\}}_{\;j\in N_{0}}$ is not unique. A common choice in the literature, also adopted in this paper, is to set

3.6 $$\beta_{j}=c_{j}F_{0}(\{j\})\;\mathrm{and}\;\omega_{j}=c_{j}(1-F_{0}(j)),\;c_{j}\in R^{+},j\in N,$$

with *c*_*j*_ denoting the strength of belief in the prior knowledge and $F_{0}(\{j\})=P_{F_{0}}(X=j)$. The name ‘strength of belief’ comes from the fact that, for high values of *c*_*j*_, it will be difficult for the posterior distribution to deviate from the prior distribution, except with large amounts of data. On the contrary, when *c*_*j*_ → 0, equation (3.1) reduces to the KM estimator of Cox & Oakes [31], which is unaffected by the choice of *F*_0_.

Finally, observe that the roles of the strength of belief parameters *c*_*j*_ and of the reinforcement parameter *r* are actually opposite. It is, therefore, possible to fix one of them and just work with the remaining one.

### 3.2. The bivariate RUP of Bulla *et al.* [2]

As mentioned in §3.1, a RUP is a one-dimensional process. In order to cope with two dimensions, a bivariate extension (B-RUP) was first proposed in [2], using a one-factor construction.

Assume we have a sample of bivariate LTRC observations of the form $\left((X_{n}^{*},T_{n}^{X},\delta_{n}^{X}),(Y_{n}^{*},T_{n}^{Y},\delta_{n}^{Y})\right)$ about two variables of interest *X* and *Y*. As before, $T_{n}^{X}$ and $T_{n}^{Y}$ are the truncation variables for *X* and *Y*, respectively. A simple way of modelling the dependence between *X* and *Y* is to consider a one-factor construction, based on three independent components: one common and two idiosyncratic factors for *X* and *Y*. Thus, let *A*, *B* and *C* be independent r.v.s and set

3.7 $$\begin{array}{cc} & X=A+B\\ \mathrm{and}\;\;\; & Y=A+C. \end{array}\}$$

The dependence between *X* and *Y* relies entirely on *A*, hence, conditioned on this common process, *X* and *Y* are independent. A straightforward calculation yields

3.8 Cov(X,Y|A,B,C)=Var(A).

Therefore, the dependence between *X* and *Y* is linear and non-negative.

The idea of Bulla *et al.* [2] was to model the distributions of *A*, *B* and *C* with RUPs using equation (3.1) in order to exploit the properties of the RUP and avoid the caveats of bivariate non-parametric estimators already mentioned in §1. The resulting joint distribution *F*_*XY*_ of *X* and *Y* is then obtained through convolutions. We refer to Bulla *et al.* [2] for a detailed explanation of the many probabilistic properties of *F*_*XY*_. For our purposes, the most relevant feature of the model is the one-factor construction. As we will see in §4, this will enable us to derive a simple and efficient iterative method to estimate *F*_*XY*_.

**Remark.**

The variables *A*, *B* and *C* are artificially created, and therefore there are no observations of these variables in practice. This poses the problem of estimating the distributions of *A*, *B* and *C* given observations of *X* and *Y*. We show in §4 that this can be considered as a type of incomplete data problem, and thus suitable for the EM and ER algorithms.

## 4. The expectation–maximization and the expectation-reinforcement algorithms

The EM algorithm was introduced in [17] to unify several, seemingly different, methodologies that extract information from incomplete data (see, for example, [38]). From a mathematical point of view, the algorithm computes the expectation of the complete log-likelihood *L*(***x***|***y***, *θ*) at each iteration, conditioned on the observed incomplete data ***y***, and the estimates of the parameters from the previous iteration *θ*. Then, it maximizes this expectation in order to find the optimal parameters θ* for the next iteration. This iteration procedure is repeated until some stopping criterion is met. In the context of this article, the incomplete data corresponds to the triplets $\left((X_{n}^{*},T_{n}^{X},\delta_{n}^{X}),(Y_{n}^{*},T_{n}^{Y},\delta_{n}^{Y})\right)$, while the complete data would be uncensored observations (***A***_*n*_, ***B***_*n*_, ***C***_*n*_) of the processes that conform the B-RUP. The set of parameters *θ* thus corresponds to the distributions of *A*, *B* and *C*.

Before explaining the details of the proposed methodology, we introduce some notation to facilitate the reading:

4.1 $$p_{X}(x)\;:=P(X=x|\theta ),$$

and

4.2 $$p_{X}^{\left[k\right]}(x)\;:=P(X=x|\theta^{\left[k\right]}),$$

where *θ* is again the set of parameters and the superscript *k* the iteration number in the EM algorithm. We also introduce the survival function $S_{X}(x)\;:=P(X>x|\theta )$, and $S_{X}^{\left[k\right]}(x)$ analogously.

As mentioned in §3, the main idea of the B-RUP is to model the distributions of (*A*, *B*, *C*) as individual, independent RUPs. In the following sections, we apply a similar idea, and define the distributions of (*A*, *B*, *C*) in terms of urns. In §4.2, it is then shown how the resulting models are related to the original RUP model.

Since the variables (*A*, *B*, *C*) are independent, we start defining the urn distribution for variable *A*, with the distributions for variables *B* and *C* defined analogously.

Let *A* be a r.v. on the positive integers such that

4.3 $$S_{A}(a)=\prod_{\;j=0}^{a}\frac{G_{j}^{A}}{N_{j}^{A}},\;a\in N_{0},$$

with the convention $\prod_{\;j=0}^{a}=1$ for *a* < 0. Following the urn representation of §3, $G_{j}^{A}$ denotes the number of green balls in urn *U*_*j*_, and $N_{j}^{A}$ the total number of balls in the same urn. These pairs ${\left(G_{j}^{A},N_{j}^{A}\right)}_{\;j\in N_{0}}$ are the variables we wish to calibrate via the EM algorithm. In other words, $\theta ={\left(G_{j}^{A},N_{j}^{A},G_{j}^{B},N_{j}^{B},G_{j}^{C},N_{j}^{C}\right)}_{\;j\in N_{0}}$. However, in the presence of left-truncation *θ* needs to be enlarged to account for the truncation variables. This is explained in §4.1.

**Remark.**

Defining the pair $\left(G_{i}^{A},N_{i}^{A}\right)$ is not really necessary. Given equation (4.3), it is the ratio between $G_{i}^{A}$ and $N_{i}^{A}$ that characterizes the distribution of *A*. The pair is relevant, however, in the context of §4.2, as this distinction is what allows the inclusion of prior knowledge and a complete analogy with the RUP.

### 4.1. Expectation–maximization for LTRC data

For simplicity, we assume first that there is no left-truncation. That is, the observations are the pairs $\left(X_{n},\delta_{n}^{X}\right)$ and $\left(Y_{n},\delta_{n}^{Y}\right)$, and we want to estimate the distributions of (*A*, *B*, *C*). Due to the one-factor construction of equation (3.7), we can write the log-likelihood of (*A*, *B*, *C*) as the sum of the log-likelihoods of *A*, *B* and *C*:

4.4 $$\log P(A=a,B=b,C=c)=\log p_{A}(a)+\log p_{B}(b)+\log p_{C}(c).$$

We present only the results for *A*, with the results for *B* and *C* following analogously. The expected complete log-likelihood of *A* at the (*k* + 1)th iteration, given a single observation (*x*, *y*, *δ*^*X*^, *δ*^*Y*^) of (*X*, *Y*) and the estimates from the *k*th iteration, is given by

4.5 $$L_{A}(\theta^{\left[k+1\right]}|\theta^{\left[k\right]})=\sum_{a=0}^{\infty }\log p_{A}^{\left[k+1\right]}(a)\;p_{A}^{\left[k\right]}(a|x,y,\delta^{X},\delta^{Y}),$$

where x∧y=min(x,y),

4.6 $$p_{A}(a|x,y,1,1)=\frac{\;p_{A}(a)p_{B}(x-a)p_{C}(y-a)}{p_{XY}(x,y)},$$

4.7 $$p_{A}(a|x,y,0,1)=\frac{\;p_{A}(a)S_{B}(x-a)p_{C}(y-a)}{P(X>x,Y=y)},$$

4.8 $$p_{A}(a|x,y,1,0)=\frac{\;p_{A}(a)p_{B}(x-a)S_{C}(y-a)}{P(X>x,Y=y)},$$

4.9 $$p_{A}(a|x,y,0,0)=\frac{\;p_{A}(a)S_{B}(x-a)S_{C}(y-a)}{P(X>x,Y>y)},$$

4.10 $$\mathrm{and}p_{XY}(x,y)=\sum_{a=0}^{x\wedge y}p_{A}(a)p_{B}(x-a)p_{C}(y-a).$$

**Remark.**

The lower limit of the summation in equation (4.10) is zero because we work with non-negative processes. The upper limit is also a consequence of non-negativity and the fact that *A* cannot be bigger than *X* or *Y*, due to equation (3.7).

The next step is to compute the derivatives of equation (4.5) with respect to *θ*^[*k*+1]^ and set them equal to zero to obtain the values of the next iteration. Given equation (4.3), it is easy to see that:

4.11 $$\frac{\partial \log p_{A}(a)}{\partial G_{j}^{A}}=\left\{ \begin{array}{cc} 0 & \text{if }j>a\\ \frac{-1}{N_{j}^{A}-G_{j}^{A}} & \text{if }j=a\\ \frac{1}{G_{j}^{A}} & \text{if }j<a. \end{array}\right.$$

Combining equations (4.11) and (4.5) yields

4.12 $$\frac{\partial L_{A}(\theta^{\left[k+1\right]}|\theta^{\left[k\right]})}{\partial G_{j}^{[k+1],A}}=\frac{S_{A}^{\left[k\right]}(j|x,y,\delta^{X},\delta^{Y})}{G_{j}^{[k+1],A}}-\frac{\;p_{A}^{\left[k\right]}(j|x,y,\delta^{X},\delta^{Y})}{N_{j}^{[k+1],A}-G_{j}^{[k+1],A}}.$$

Setting this last expression equal to zero and solving for $G_{j}^{[k+1],A}$ yields the value for the next iteration:

4.13 $$\frac{G_{j}^{[k+1],A}}{N_{j}^{[k+1],A}}=\frac{S_{A}^{\left[k\right]}(j|x,y,\delta^{X},\delta^{Y})}{S_{A}^{\left[k\right]}(j-1|x,y,\delta^{X},\delta^{Y})},$$

where we have used *S*_*A*_(*j*) + *p*_*A*_(*j*) = *S*_*A*_(*j* − 1), since *A* is discrete with jumps of size one.

In the case of *n* observations, equation (4.13) becomes:

4.14 $$\frac{G_{j}^{[k+1],A}}{N_{j}^{[k+1],A}}=\frac{\sum_{i=1}^{n}S_{A}^{\left[k\right]}(j|x_{i},y_{i},\delta_{i}^{X},\delta_{i}^{Y})}{\sum_{i=1}^{n}S_{A}^{\left[k\right]}(j-1|x_{i},y_{i},\delta_{i}^{X},\delta_{i}^{Y})}.$$

The solutions for $G_{j}^{B}$ and $G_{j}^{C}$ are completely analogous, and their respective formulae are provided below:

4.15 $$p_{B}(b|x,y,0,1)=\frac{\;p_{B}(b)\sum_{a=x-b+1}^{y}p_{A}(a)p_{C}(y-a)}{P(X>x,Y=y)},$$

4.16 $$p_{B}(b|x,y,1,0)=\frac{\;p_{B}(b)p_{A}(x-b)S_{C}(y-x+b)}{P(X=x,Y>y)},$$

4.17 $$p_{B}(b|x,y,0,0)=\frac{\;p_{B}(b)\sum_{a=x-b+1}^{\infty }p_{A}(a)S_{C}(y-a)}{P(X>x,Y>y)},$$

4.18 $$p_{C}(c|x,y,1,0)=\frac{\;p_{C}(c)\sum_{a=y-c+1}^{x}p_{A}(a)p_{B}(x-a)}{P(X=x,Y>y)},$$

4.19 $$p_{C}(c|x,y,0,1)=\frac{\;p_{C}(c)p_{A}(y-c)S_{B}(x-y+c)}{P(X>x,Y=y)},$$

4.20 $$\mathrm{and}\;\;p_{C}(c|x,y,0,0)=\frac{\;p_{C}(c)\sum_{a=y-c+1}^{\infty }p_{A}(a)S_{B}(x-a)}{P(X>x,Y>y)}.$$

The final step is to consider bivariate left-truncation. In this case, we observe (*x*, *y*, *δ*^*X*^, *δ*^*Y*^, *t*^*X*^, *t*^*Y*^) if (*x* ≥ *t*^*X*^) and (*y* ≥ *t*^*Y*^) and nothing otherwise. The complete data, therefore, consists of observations (*x*, *y*, *δ*^*X*^, *δ*^*Y*^, *t*^*X*^, *t*^*Y*^) regardless of whether (*x* ≥ *t*^*X*^) and (*y* ≥ *t*^*Y*^) is verified. If we denote the truncation event by A, the expectation of the complete log-likelihood of *A* for a sample of size *n* becomes

4.21 $$\begin{array}{rl} L_{A}(\theta^{\left[k+1\right]}|\theta^{\left[k\right]}) & =\sum_{i=1}^{n}\sum_{a=0}^{\infty }\log p_{A}^{\left[k+1\right]}(a)p_{A}^{\left[k\right]}(a|x_{i},y_{i},\delta_{i}^{X},\delta_{i}^{Y})+(M^{\left[k\right]}-n)\sum_{a=0}^{\infty }\log p_{A}^{\left[k+1\right]}(a)p_{A}^{\left[k\right]}(a|A), \end{array}$$

where, in general,

4.22 $$M^{\left[k\right]}=\frac{n}{p^{\left[k\right]}(A^{\;c})}$$

is the total number of samples. That is, the number of observations (*n*) plus the unobserved samples due to left-truncation.

A consists of three different events: (*T*^*X*^ ≤ *X*, *T*^*Y*^ > *Y*), (*T*^*X*^ > *X*, *T*^*Y*^ ≤ *Y*) and (*T*^*X*^ > *X*, *T*^*Y*^ > *Y*). On the other hand, the complementary of A, i.e. the observation event, is defined as

4.23 $$A^{\;c}=(T^{X}\leq X,T^{Y}\leq Y).$$

Given that

4.24 $$p(\cdot )=p(\cdot |A)p(A)+p(\cdot |A^{\;c})p(A^{\;c}),$$

it is straightforward to compute $p_{A}(a|A^{c})$ and then use equation (4.24) to condition on A. For that purpose, we also need the probability of the observation event, which can be computed as

4.25 $$p(A^{\;c})=\sum_{x=0}^{\infty }\sum_{y=0}^{\infty }p_{XY}(x,y)P(T^{X}\leq x,T^{Y}\leq y),$$

where *p*_*XY*_(*x*, *y*) is defined as in equation (4.10) and, using the truncation assumptions of §2,

4.26 $$p(T^{X}\leq x,T^{Y}\leq y)=\sum_{t=0}^{x}p_{T^{X}}(t)p_{\epsilon }(\epsilon \leq y-t),$$

with $p_{T^{X}}$ and $p_{\epsilon }$ the probability distributions of *T*^*X*^ and ϵ, respectively.

**Remark.**

From equation (4.25), it is clear that we need the distribution of the truncation variables (*T*^*X*^, *T*^*Y*^). This distribution can be computed from the marginal distributions of *T*^*X*^ and ϵ. We assume that these are also urn distributions as in equation (4.3). Therefore, as mentioned previously in this section, the total set of parameters to be estimated becomes $\theta ={\left(G_{j}^{A},N_{j}^{A},G_{j}^{B},N_{j}^{B},G_{j}^{C},N_{j}^{C},G_{j}^{T^{X}},N_{j}^{T^{X}},G_{j}^{\epsilon },N_{j}^{\epsilon }\right)}_{\;j\in N_{0}}$ in the presence of left-truncation.

The summation over *i* in equation (4.21) does not depend on the truncation event, and thus it can be evaluated using equations (4.6)–(4.9). On the other hand, the truncation component in equation (4.21) can be computed using equations (4.24) and (4.25) and

4.27 $$p_{A}(a|A^{\;c})=\frac{\;p_{A}(a)}{p(A^{\;c})}\sum_{b=0}^{\infty }\sum_{c=0}^{\infty }p_{B}(b)p_{C}(c)P(T^{X}\leq a+b,T^{Y}\leq a+c).$$

The optimal configuration *θ*^[*k*+1]^ of the next iteration is then obtained by applying the derivative operator to equation (4.21) and then using equation (4.11). This yields

4.28 $$\frac{G_{j}^{[k+1],A}}{N_{j}^{[k+1],A}}=\frac{\sum_{i=1}^{n}S_{A}^{\left[k\right]}(j|x_{i},y_{i},\delta_{i}^{X},\delta_{i}^{Y})+(M^{\left[k\right]}-n)S_{A}^{\left[k\right]}(j|A)}{\sum_{i=1}^{n}S_{A}^{\left[k\right]}(j-1|x_{i},y_{i},\delta_{i}^{X},\delta_{i}^{Y})+(M^{\left[k\right]}-n)S_{A}^{\left[k\right]}(j-1|A)}.$$

The results for *B* and *C* are completely analogous, and the necessary formulae to compute the estimates are provided in equations (4.15)–(4.20), (4.29) and (4.30). For the truncation variables, equation (4.28) is actually less involved, since these variables do not suffer from censoring. The conditional probabilities are given in equations (4.31) and (4.32), and the estimators in equations (4.33) and (4.34), respectively.

4.29 $$p_{B}(b|A^{\;c})=\frac{\;p_{B}(b)}{p(A^{\;c})}\sum_{a=0}^{\infty }\sum_{c=0}^{\infty }p_{C}(c)p_{A}(a)P(T^{X}\leq a+b,T^{Y}\leq a+c),$$

4.30 $$p_{C}(c|A^{\;c})=\frac{\;p_{C}(c)}{p(A^{\;c})}\sum_{a=0}^{\infty }\sum_{b=0}^{\infty }p_{B}(b)p_{A}(a)P(T^{X}\leq a+b,T^{Y}\leq a+c),$$

4.31 $$p_{T^{X}}(t|A^{\;c})=\frac{\;p_{T^{X}}(t)}{p(A^{\;c})}\sum_{e=-\infty }^{\infty }p_{\epsilon }(e)P(X\geq t,Y\geq t+e),$$

4.32 $$p_{\epsilon }(e|A^{\;c})=\frac{\;p_{\epsilon }(e)}{p(A^{\;c})}\sum_{t=0}^{\infty }p_{T^{X}}(t)P(X\geq t,Y\geq t+e),$$

4.33 $$\frac{G_{j}^{[k+1],T^{X}}}{N_{j}^{[k+1],T^{X}}}=\frac{\sum_{i=1}^{n}1_{\left\{t_{i}>j\right\}}+(M^{\left[k\right]}-n)\;S_{T^{X}}^{\left[k\right]}(j|A)}{\sum_{i=1}^{n}1_{\left\{t_{i}\geq j\right\}}+(M^{\left[k\right]}-n)\;S_{T^{X}}^{\left[k\right]}(j-1|A)},$$

4.34 $$\mathrm{and}\;\;\frac{G_{j}^{[k+1],\epsilon }}{N_{j}^{[k+1],\epsilon }}=\frac{\sum_{i=1}^{n}1_{\left\{e_{i}>j\right\}}+(M^{\left[k\right]}-n)S_{\epsilon }^{\left[k\right]}(j|A)}{\sum_{i=1}^{n}1_{\left\{e_{i}\geq j\right\}}+(M^{\left[k\right]}-n)\;S_{\epsilon }^{\left[k\right]}(j-1|A)},$$

where $T_{n}^{X}=(t_{1},\ldots ,t_{n})$ and $\epsilon_{n}=(e_{1},\ldots ,e_{n})$ are the observed samples of *T*^*X*^ and ϵ, respectively.

### 4.2. The expectation-reinforcement algorithm

The ER algorithm aims at combining the reinforcement mechanism of RUPs with the EM algorithm offering the possibility of embedding prior knowledge and experts’ judgements into the estimates. This feature is particularly useful in the modelling of extreme events [39], epistemic uncertainty [40,41], or when there are possible biases in the sample, as is the case with LTRC data. For example, it is well-known that the left-truncation effect produces a positive bias in the average lifetime of an individual in medical and survival studies, making the average lifetime look larger than it actually is. An expert familiar with this phenomenon can correct this bias by selecting a prior distribution with a smaller expected value than the one observed in-sample. We illustrate an example of such bias in §5.2, where we analyse the consequences of ignoring left-truncation in the estimators. In the context of extreme events that, due to their nature, are rarely present or not present at all in the data, this bias can also be corrected by choosing a prior distribution that gives a larger weight to these events. For example, due to the right-censoring effect, it is extremely rare to observe unusually large expected lifetimes in survival studies. Hence, in order to incorporate such unusual lifetimes in the posterior distribution, this information can be extracted, for example, from mortality tables and included in the prior distribution, correcting the final estimates.

Moreover, non-parametric estimators usually suffer from overfitting [42]. Such a problem occurs when the model calibrates too well to the sample data, making the procedure highly sensitive to small variations in the sample properties, and thus reducing its predictive power out-of-sample. From the bias-variance trade-off point of view, non-parametric estimators tend to have a very small bias, as they capture all the features of the dataset. On the other hand, their variance can be considerably large, since their parameters are very sensitive to small changes in the observations. By embedding the reinforcement mechanism of RUPs into the EM algorithm, this trade-off can be controlled as follows: for high strengths of belief (or a very small reinforcement), the posterior distribution will not be affected by the observations and will, therefore, tend to coincide with the prior distribution. In the opposite case, with almost zero strength of belief (or strong reinforcement), the posterior will adapt to the data as much as possible. In the first scenario, the variance of the model with respect to different datasets is zero, but the bias will be arbitrarily high depending on the choice of the prior distribution. In the second scenario, the bias should be considerably small, but the model will be very sensitive to small changes in the data, resulting in a large variance. Thus the trade-off can be balanced by choosing intermediate values of the strength of belief and reinforcement parameters. Nevertheless, non-parametric estimators also have other ways of dealing with the bias-variance trade-off, such as the stopping criteria of the optimization algorithm, or the use of smoothing kernels on the final estimates (see [20]).

The goal of the ER algorithm is to combine a prior distribution, given by the pairs ${\left\{\beta_{j},\omega_{j}\right\}}_{\;j\in N_{0}}$ defined in equation (3.6), with the estimates obtained from the EM algorithm, so as to obtain a posterior distribution that mixes experts’ knowledge and data.

In the following, we illustrate how to include a prior distribution to the estimates in equation (4.28). The same procedure can be applied analogously to the other variables $\left(B,C,T^{X},\epsilon \right)$.

Let the pairs ${\left\{\beta_{j}^{A},\omega_{j}^{A}\right\}}_{\;j\in N_{0}}$ define a prior distribution through equation (3.6). Next, we follow the steps described in §4.1 to obtain the EM estimates of *A*. Then, we define the posterior configuration of *A* using the following expression

4.35 $$G_{j}^{A}=\omega_{j}^{A}+r\left[\sum_{i=1}^{n}S_{A}^{\mathrm{EM}}(j|x_{i},y_{i},\delta_{i}^{X},\delta_{i}^{Y})+(M^{\mathrm{EM}}-n)\;S_{A}^{\mathrm{EM}}(j|A)\right],$$

and

4.36 $$N_{j}^{A}=\beta_{j}^{A}+\omega_{j}^{A}+r\left[\sum_{i=1}^{n}S_{A}^{\mathrm{EM}}(j-1|x_{i},y_{i},\delta_{i}^{X},\delta_{i}^{Y})+(M^{\mathrm{EM}}-n)\;S_{A}^{\mathrm{EM}}(j-1|A)\right],$$

where *r* is the reinforcement parameter defined in §3.1, and the EM superscript indicates the estimates obtained with the EM algorithm.

By giving different values to the reinforcement and belief parameters,^2^ we can control the weight of each component on the estimates. Similarly to the behaviour shown in §3.1, when the strength of belief tends to zero, we recover the estimates of the EM algorithm, while if the reinforcement tends to zero instead, the posterior distribution equals the prior distribution.

The pseudocode to implement the ER algorithm can be found in algorithm 1. It refers to the equations related to the estimates of variable *A*, but analogous formulae for the other variables can be found in §4.1.


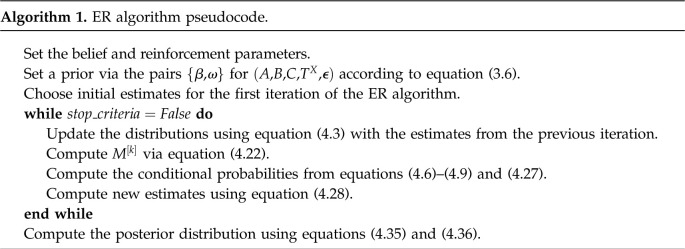


### 4.3. Error analysis

In this section, we comment on the numerical errors incurred when approximating the infinite summations appearing in the formulae of §4.1.

First, we start considering the case without left-truncation. This is the same as computing equation (4.28) (and the analogous formulae for *B* and *C*) with *M*^[*k*]^ = *n*. In that case, the formulae to be computed are equations (4.6)–(4.9). For that purpose, the distribution of (*X*, *Y*) must be known for all observed values. Therefore, there is no need to compute the distribution of (*X*, *Y*) for values outside of the observation range. Denoting by *x*_max_ and *y*_max_ the maximum observed values of *X* and *Y*, respectively, and taking into account equation (4.10), it is clear that the distributions of *A*, *B* and *C* do not need to be computed for values greater than (*x*_max_ ∨ *y*_max_), *x*_max_ and *y*_max_, respectively, where ∨ is the maximum operator.

In the presence of left-truncation, equations (4.31) and (4.32) require the entire survival distribution of (*X*, *Y*), hence the distribution of (*X*, *Y*) for values greater than *x*_max_ and *y*_max_ is also needed. We assume that the distributions of *T*^*X*^ and ϵ are limited to the intervals $\left[0,T_{\max}^{X}\right]$ and $\left[\epsilon_{\min},\epsilon_{\max}\right]$, respectively, since there are no observations outside of these ranges. Next, assume we compute the distribution of (*X*, *Y*) only in the domain [0, *x*_*M*_] × [0, *y*_*M*_]. Then equation (4.31)—the analysis of equation (4.32) is analogous, and therefore omitted—can be approximated as

4.37 $$p_{T^{X}}(t|A^{\;c})\approx {\hat{\;p}}_{T^{X}}(t|A^{\;c})\;:=\frac{\;p_{T^{X}}(t)}{p(A^{\;c})}\sum_{e=\epsilon_{\min}}^{\epsilon_{\max}\wedge (y_{M}-t)}p_{\epsilon }(e)P(X\geq t,Y\geq t+e),$$

where, due to the nature of the left-truncation effect, $T_{\max}^{X}\leq x_{\max}$, and thus only the upper bound *y*_*M*_ affects equation (4.37).

The error incurred by this approximation is given by

4.38 $$p_{T^{X}}(t|A^{\;c})-{\hat{\;p}}_{T^{X}}(t|A^{\;c})=\frac{\;p_{T^{X}}(t)}{p(A^{\;c})}\sum_{e=y_{M}-t+1}^{\epsilon_{\max}}p_{\epsilon }(e)P(X\geq t,Y\geq t+e),$$

which can be upper-bounded by

4.39 $$p_{T^{X}}(t|A^{\;c})-{\hat{\;p}}_{T^{X}}(t|A^{\;c})\leq \frac{\;p_{T^{X}}(t)}{p(A^{\;c})}S_{\epsilon }(y_{M}-t)P(X\geq t,Y\geq y_{M})=:\Omega_{y_{M}}(t),$$

for $t\geq y_{M}-\epsilon_{\max}$ and zero otherwise.

For large enough values of *y*_*M*_, the convergence of equation (4.39) towards zero with respect to *y*_*M*_ depends mostly on the right-tail of the distribution of *Y*. This, in turn, depends on the differentiability of the marginal distribution of *Y*. If the first *k* derivatives are nonzero, then the order of convergence is $O(y_{M}^{-k-1})$. For infinitely differentiable distributions, the order of convergence is exponential, i.e. $O(\;e^{-\gamma y_{M}^{r}})$, for some *γ*, *r* > 0.

**Figure 2.**
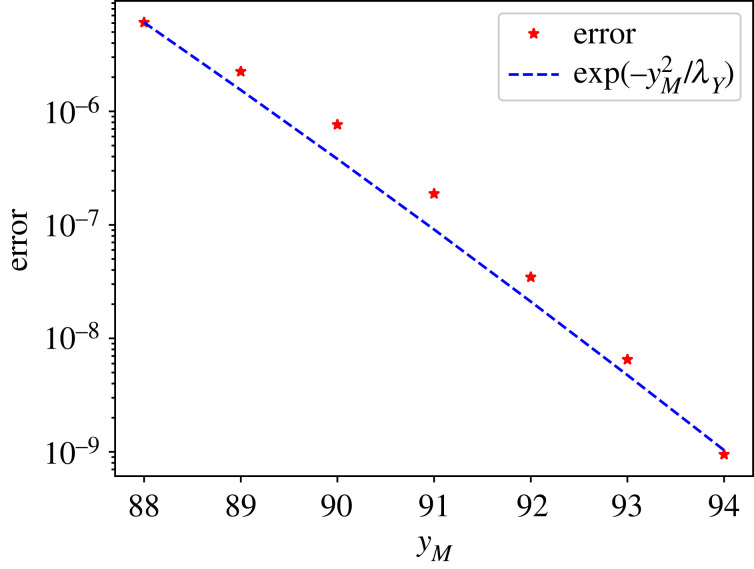
Empirical versus theoretical convergence of $\Omega_{y_{M}}$ in the Poisson simulated example.

We illustrate this using the same dataset as in §5.1, in which *Y* follows a Poisson distribution with parameter *λ*_*Y*_ = 65. In figure 2, we compute $\Omega_{y_{M}}\;:=\sum_{t=0}^{T_{\max}^{X}}\Omega_{y_{M}}(t)$ for several values of *y*_*M*_ and compare it with the theoretical convergence of the Poisson distribution, which is $O(\;e^{-y_{M}^{2}/\lambda_{Y}})$. The results confirm the exponential convergence of $\Omega_{y_{M}}$ in the Poisson example. For reference, in this example *y*_max_ = 85, thus in this case it is not necessary to choose *y*_*M*_ much larger than *y*_max_ to obtain good approximations. On the other hand, if the differentiability of the distribution of (*X*, *Y*) is expected to be small, large values of *y*_*M*_ should be used to guarantee convergence. In particular, choosing $y_{M}=T_{\max}^{X}+\epsilon_{\max}$ guarantees that $\Omega_{y_{M}}=0$.

## 5. Numerical results

We perform several numerical experiments to test the performance of the algorithms developed in §4. We start in §5.1 with an example that uses simulated data for which the reference distributions are known. Then, in §5.2, we analyse a Canadian dataset of coupled lifetimes widely used in the literature of actuarial sciences in the context of joint annuity evaluation [20–22]. This dataset is known for its complexity, due to the strong presence of censoring and truncation. Since the reference distribution for this problem is not known, we compare the results of the ER algorithm with the Frank copula of Frees *et al.* [21], which has offered satisfactory results. Moreover, we compare our results with those of Arias & Cirillo [20], where a B-RUP was first used, but only assuming right-censoring, that is, without left-truncation. This comparison allows us to study the impact of left-truncation on the empirical dataset, and to illustrate the advantages of the ER algorithm over the Markov chain Monte Carlo approach used in [20].

**Remark.**

Note that, in the context of non-parametric estimators, it is not possible to obtain information about the distribution of (*X*, *Y*) for values outside the intervals $\left(\max(X_{n}^{*}|\delta_{n}^{X}=1)\geq X\geq \min(X_{n}^{*}|\delta_{n}^{X}=1)\right)$ and $\left(\max(Y_{n}^{*}|\delta_{n}^{Y}=1)\geq Y\geq \min(Y_{n}^{*}|\delta_{n}^{Y}=1)\right)$, where the condition ***δ***_*n*_ = 1 implies that we refer to uncensored observations. In other words, the final estimates correspond to the distribution of (*X*, *Y*) within those intervals.

In order to study the impact of the prior distribution on the final estimates, we distinguish between two scenarios: *low* strength of belief and *high* strength of belief. In the remainder of this work, we denote these scenarios by ER^*l*^ and ER^*h*^, respectively. The values for the belief and reinforcement parameters for each case can be found in table 1. We assume the same strengths of belief for all urns. In practice, it could be interesting to attach different strengths of belief to each urn. For example, for the urns associated with the bulk of the data we could use low strengths of belief, and higher values for areas were observations are sparse. Regarding the stopping criterion, we have chosen the absolute value of the relative difference between the incomplete log-likelihood (see equation (2.3)) of the current and previous iterations. When this quantity is below a certain threshold, or the number of iterations reaches a prespecified limit, the algorithm stops. For all experiments considered in this paper, we have chosen the threshold to be 10^−9^ and the maximum number of iterations as 10^4^.

**Table 1.** Proposed scenarios defined by the values of the belief and reinforcement parameters. The values are used throughout §5. The superscripts in the ER columns denote ‘low’ and ‘high’, respectively, referring to the weight of the belief parameters. Here *r* is again the reinforcement parameter and *c* is the strength of belief parameter.

	EM	ER^*l*^	ER^*h*^
*r*	—	10^4^	1
*c*	0	1	10

**Remark.**

The algorithm was implemented in C++ using the g++ compiler (v. 9.4.0) and is freely available in the GitHub repository: https://github.com/LuisSouto/Expectation-Reinforcement. Experiments were run using an Intel(R) Core(TM) i7-7700HQ CPU @ 2.80GHz processor.

### 5.1. Example with simulated data

The first experiment we consider consists of a simulated dataset where the observations come from a one-factor model as in equation (3.7). Hence, the model assumptions are verified and it is expected that the ER algorithm yields reasonable results. We refer to this experiment as the Poisson simulated example, since we assume Poisson distributions for all variables involved. Further experiments using a combination of discrete uniform distributions, Beta-Binomial distributions and Negative Hypergeometric distributions have also yielded positive results (not shown here) and are available upon request.

The data is generated using the following distributions: *A* ∼ Poi(40), *B* ∼ Poi(20), *C* ∼ Poi(25), *T* ∼ Poi(70), ϵ∼Poi(7)−5 and Δ ∼ Poi(2), where Poi(*λ*) denotes a Poisson distribution with parameter *λ*. Therefore, in this case *X* ∼ Poi(60) and *Y* ∼ Poi(65), respectively, and the correlation between them is approximately 0.64. The reader is referred to §2.1 for a review of the censoring and truncation assumptions. The sample consists of 10^4^ pairs of LTRC triplets $\left((X_{n}^{*},T_{n}^{X},\delta_{n}^{X}),(Y_{n}^{*},T_{n}^{Y},\delta_{n}^{Y})\right)$. The probability of truncation is approximately 82%, and the number of unobserved samples due to truncation is 53 096. Within the observed sample, the percentage of double censored observations is 65.73% and the percentage of observations with at least *X* or *Y* censored is 92.23%. Therefore, this is a dataset with both a high probability of censoring and truncation.

Following algorithm 1, we start by choosing a prior distribution for each variable. In this example, we have chosen $F_{0}^{A}=\mathrm{Poi}(20)$, $F_{0}^{B}=\mathrm{Poi}(20)$, $F_{0}^{C}=\mathrm{Poi}(20)$, $F_{0}^{T}=\mathrm{Poi}(50)$ and $F_{0}^{\epsilon +10}=\mathrm{Poi}(10)$, where $F_{0}^{\epsilon +5}=\mathrm{Poi}(10)$ implies that ϵ∼Poi(10)−5 according to the prior distribution. The value −5 in the distribution of ϵ was inferred from its minimum observed value. The next step is the choice of the initial estimates. Both the ER and EM algorithms are local optimization algorithms, and whether a global or local minimum is reached, therefore, depends on the choice of the initial estimate. In practice, it is common to use several initial estimates to overcome this problem (see [43] and references therein), but we use the prior distributions as initial estimates for simplicity.

**Remark.**

All results regarding this dataset are conditioned on 45 ≤ *X* ≤ 82 and 49 ≤ *Y* ≤ 84, since these are the maximum and minimum uncensored values observed. Moreover, the computational cost of the ER algorithm under the specified stopping criterion and hardware was 24 s.

**Figure 3.**
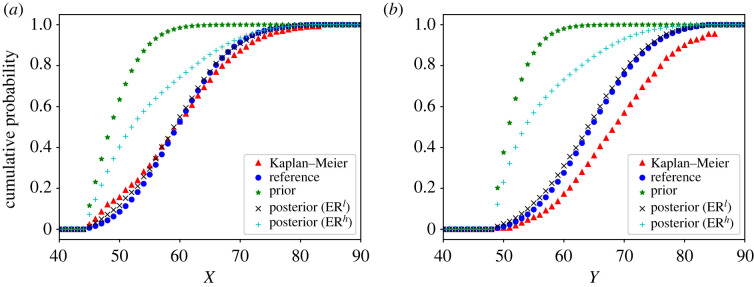
Fitting of the marginal distributions of *X* (*a*) and *Y* (*b*) in the Poisson simulated example.

In figure 3, we present the ER estimates of the marginal distributions of *X* and *Y*. For comparison purposes, the KM estimator and the prior distribution are also shown. Note that the KM estimator is clearly biased with respect to the reference distribution due to the presence of bivariate left-truncation. The plots also illustrate the impact of the strength of belief parameters on the final estimates. For low strengths of belief (ER^*l*^), the distribution only takes the data into account, and matches well with the reference distribution. For high strengths of belief (ER^*h*^), the distribution is more influenced by the prior knowledge, specially in areas with fewer observations.

**Figure 4.**
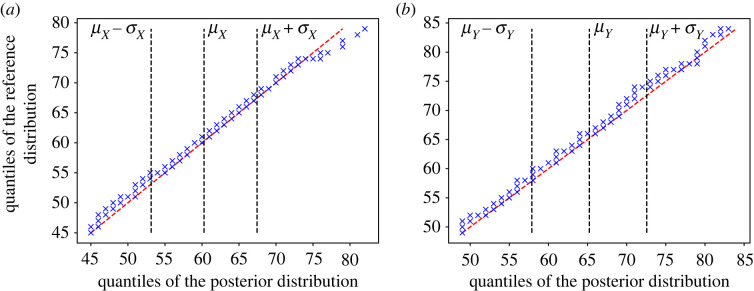
QQ-plot comparing the ER^*l*^ posterior and reference distributions. The dashed lines correspond to the mean (*μ*) and one standard deviation (*σ*) from the mean in each direction. (*a*) QQ-plot: *X* and (*b*) QQ-plot: *Y*.

A further analysis of this comparison can be found in figure 4, where we show the QQ-plots for the ER^*l*^ marginal distributions against the reference ones, and table 2, which contains the results of permutation tests for the mean values and the variances of *X* and *Y* also obtained with the ER^*l*^ estimates. The QQ-plots were generated using two samples of size 500 from each marginal of the ER^*l*^ posterior distribution, and comparing them with a sample of the same size from the reference solution. The same samples are also used to perform the permutation tests in table 2. We use the difference in mean values as test statistics for the mean values themselves, and the difference in variances as test statistics for the variances themselves. The null hypothesis assumes that the ER^*l*^ estimates match with the reference ones with a 5% confidence threshold.

**Table 2.** Permutation test results for samples from the ER^*l*^ marginal estimators. The first column gives the value of the test statistic, the second is the *p*-value and the last column checks whether the null hypothesis is rejected or not. The number of permutations is 10^5^ in all tests.

	value	*p* (%)	*H*_0_
Mean(*X*)	0.304	53.73	do not reject
Mean(*Y*)	0.722	14.49	do not reject
Var(*X*)	−8.891	7.81	do not reject
Var(*Y*)	1.195	82.71	do not reject

**Figure 5.**
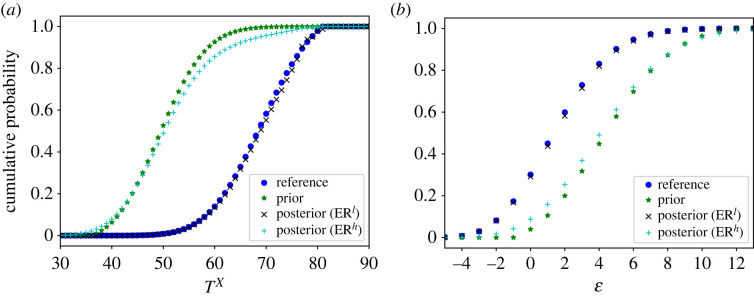
Fitting of the marginal distributions of *T*^*X*^ (*a*) and ϵ (*b*) in the Poisson simulated example.

Table 3 contains a comparison of the mean values, variances, correlation and probability of truncation obtained with each model. Compared to the reference distribution, the ER^*l*^ estimator generates the most similar values. The ER^*h*^ estimator aims for a compromise between the data and the prior distribution, which is reflected in the smaller mean values and larger variances. The KM estimator also gives reasonable results, although it is clearly biased, for example, in the mean of *Y* and the variance of *X*. Regarding the probability of truncation, the ER^*l*^ value is very close to the reference one, while the prior distribution underestimates the amount of truncation. A further comparison of the truncation variables can be seen in figure 5, where we show the marginal distributions of *T*^*X*^ and ϵ under the different models.

**Table 3.** Table with the mean values and variances of *X* and *Y*, the correlation between *X* and *Y* and the probability of truncation in the Poisson simulated example.

	mean(*X*)	mean(*Y*)	var(*X*)	var(*Y*)	corr(*X*, *Y*)	p(A)
reference	60.2875	65.2269	51.0608	53.8907	0.5939	0.8254
prior	49.8009	52.1530	15.5717	9.4091	0.1905	0.5551
ER^*l*^	59.8433	64.7188	57.5964	56.2892	0.6402	0.8382
ER^*h*^	55.1751	56.8845	72.3250	56.6427	0.7776	0.4266
KM	59.9198	68.1797	71.9409	61.5444	—	—

**Figure 6.**
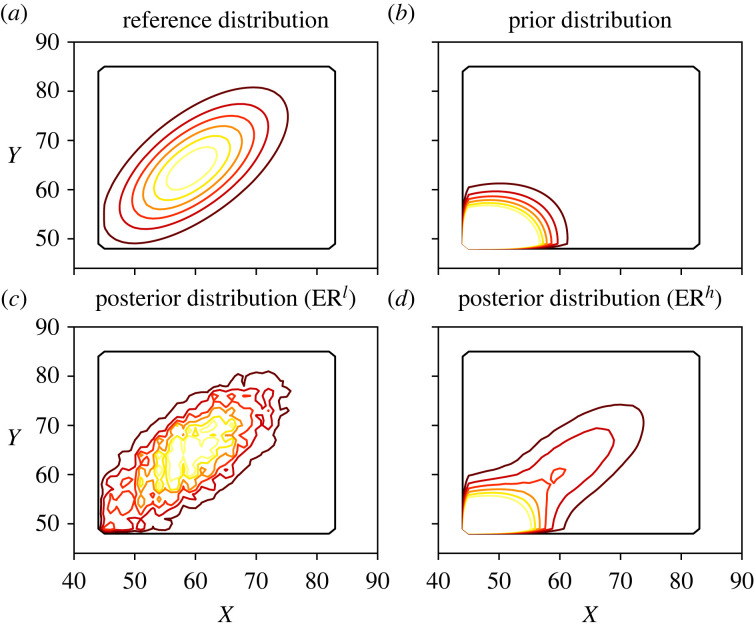
Contour plots of the reference, prior and posterior distributions in the Poisson simulated example. The levels for each curve are the same in all plots. On the upper row the reference and prior distributions are presented, while the lower row contains the ER posterior distributions with low (*a,c*) and high (*b,d*) strengths of belief.

Finally, in figure 6, a contour plot of the reference, prior and posterior distributions is presented. Consistently with the previous results, the ER^*l*^ estimator is the closest to the reference distribution, while the ER^*h*^ estimator still presents features of both the prior distribution and the observed data. Note that the curves of the ER^*l*^ estimator are not smooth compared to the reference distribution, showing signs of overfitting. As mentioned in §4.2, this could be tackled using a prior distribution, modifying the stopping criteria or by using smoothing kernels.

### 5.2. Empirical data: coupled lifetimes

In the next experiment, we consider an empirical dataset of coupled lifetimes, which is widely used in the field of joint annuity modelling [21,22]. The data consists of almost 15 000 couples of clients of a Canadian insurance company. Each couple has a joint annuity contract with the insurer. For each couple several pieces of information are available: date of contract, date of birth of the two annuitants, date of death (if observed), age at the end of the observation window, incomes, etc.

Following Luciano *et al.* [22], we remove same-sex contracts due to their scarcity in the dataset and define *X* and *Y* as the lifetime of males and females in the couple, respectively. In the same paper [22], the authors also mention that a couple may have entered into more contracts, and thus they may appear several times in the dataset. Therefore, we remove all repeated entries so that each couple is considered only once. We also remove entries where the annuitants were older than 100 years old at the beginning of the observation period. Finally, as in [21], we condition on couples that are at least 40 years old. This leaves us with a total of 11 420 male–female couples, of which only 197 are completely uncensored. Since the period of observation is, at most, 5 years, the effects of left-truncation and right-censoring are expected to be large when determining the underlying distribution. For an extended analysis of this dataset, we refer to Frees *et al.* [21] and Luciano *et al.* [22].

We analyse the effect of two different prior distributions: one that clearly underestimates the mean values and variances observed in the data (ER_1_), and another that takes this information into account to generate a good starting point in the calibration (ER_2_). For each choice of a prior distribution, we consider the same two scenarios introduced in table 1. Moreover, these results are compared with the Frank copula model defined in [21] in order to analyse the performance of the proposed estimators and with the results of Arias & Cirillo [20] to assess the impact of left-truncation on the posterior distribution.

Similar to the procedure followed in §5.1, we define first the prior and initial distributions for each variable. For simplicity, we assume that the prior and initial distributions coincide. For the first example we choose: $F_{0}^{A}=\mathrm{Poi}(20)$, $F_{0}^{B}=\mathrm{Poi}(20)$, $F_{0}^{C}=\mathrm{Poi}(20)$, $F_{0}^{T^{X}}=\mathrm{Poi}(50)$ and $F_{0}^{\epsilon -40}=\mathrm{Poi}(10)$, where the constant in ϵ−40 was inferred from the maximum age difference observed in the dataset. According to this distribution, males and females have the same average lifetime of 40 years, with a standard deviation of almost 6.5 years. Moreover, the average age difference is 30 years, with a standard deviation of approximately 3 years. For the second example we choose: $F_{0}^{A}=\mathrm{Poi}(40)$, $F_{0}^{B}=\mathrm{Poi}(40)$, $F_{0}^{C}=\mathrm{Poi}(45)$, $F_{0}^{T^{X}}=\mathrm{Poi}(70)$ and $F_{0}^{\epsilon -40}=\mathrm{Poi}(40)$. Under this choice of prior distribution, males and females have an average lifetime of 80 and 85 years, respectively, with a standard deviation of approximately 9 years. Also, the average age difference is zero, with a standard deviation of almost 6.5 years.

Regarding the copula model, Frees *et al.* [21] use Gompertz distributions for the individual lifetimes, and the Frank copula to model the dependence. The Gompertz distribution is given by

5.1 $$\text{Gomp}(x;\mu ,\sigma )=1-\exp(e^{-(\mu /\sigma )}(1-e^{\left(x/\sigma \right)})),$$

where *μ*, *σ* are the location and scale parameter, respectively.

The Frank copula is defined as

5.2 $$C(u,v;\alpha )=\frac{1}{\alpha }\log\left(1+\frac{\left(e^{\alpha u}-1)(e^{\alpha v}-1\right)}{e^{\alpha }-1}\right),$$

where *u*, *v* are the marginal distributions for the male and female annuitants, respectively, and *α* is the parameter controlling the dependence. A negative value of *α* indicates positive dependence, while *α* = 0 indicates independence [44].

We follow the same procedure of Frees *et al.* [21] to estimate the model parameters, to which we refer for all details. In table 4, the optimal parameters obtained via maximum likelihood estimation (MLE) are presented, where (*μ*_*X*_, *σ*_*X*_) are the estimates for the male annuitants, and (*μ*_*Y*_, *σ*_*Y*_) the estimates for the female annuitants. Since the value of *α* is highly negative, we expect a strong positive dependence, which justifies the use of the B-RUP model.

**Table 4.** Calibration of the Frank copula model using MLE. The subscripts *X*, *Y* refer to the marginals of the male and female annuitants, respectively.

*μ*_*X*_	*σ*_*X*_	*μ*_*Y*_	*σ*_*Y*_	*α*
84.809	9.926	87.575	7.792	−4.081

**Remark.**

All results regarding this dataset are conditioned on 51 ≤ *X* ≤ 99 and 46 ≤ *Y* ≤ 98, since these are the maximum and minimum uncensored values observed. Moreover, the computational cost of the ER algorithm under the specified stopping criteria and hardware was 21 s for scenario ER_1_ and 27 s for scenario ER_2_.

**Figure 7.**
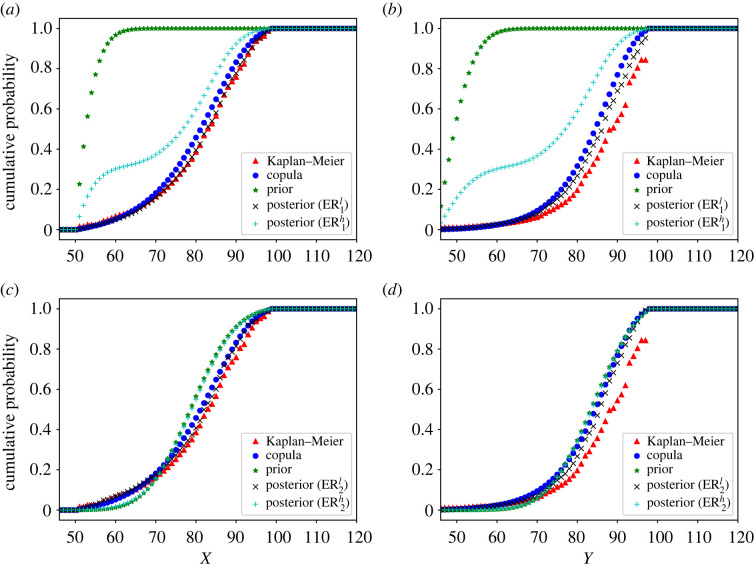
Marginal distributions of *X* and *Y* using the Canadian dataset. The upper row contains the ER_1_ scenarios and the lower row the ER_2_ scenarios. The copula and KM distributions are the same in both rows. (*a*) Marginal of *X*, (*b*) marginal of *Y*, (*c*) marginal of *X* and (*d*) marginal of *Y*.

In figure 7, we show the marginal distributions of *X* and *Y* for all scenarios and models considered. As expected, the first prior distribution is considerably different from the observations, which affects significantly the posterior distribution in scenario ${\mathrm{ER}}_{1}^{h}$. On the other hand, the posterior distribution in ${\mathrm{ER}}_{1}^{l}$ is affected only by the data, due to the choices of the reinforcement and strength of belief parameters. Note that the ${\mathrm{ER}}_{1}^{l}$ and ${\mathrm{ER}}_{2}^{l}$ estimators are almost equal, since they only depend on the observations and the choice of the initial distribution in the ER algorithm, but not on the prior distribution. However, the ${\mathrm{ER}}_{2}^{h}$ estimator is significantly different from the ${\mathrm{ER}}_{1}^{h}$ one. Since the second prior distribution was chosen based on the data, it cannot be stated that the prior distribution brings in new information in this case. Nevertheless, it can be used as a reasonable starting point in the ER algorithm and to smoothen the posterior distribution. Regarding the copula model, the distributions obtained with the Frank copula and the ER algorithm are quite similar, signalling the robustness of the results obtained with both methods.

**Figure 8.**
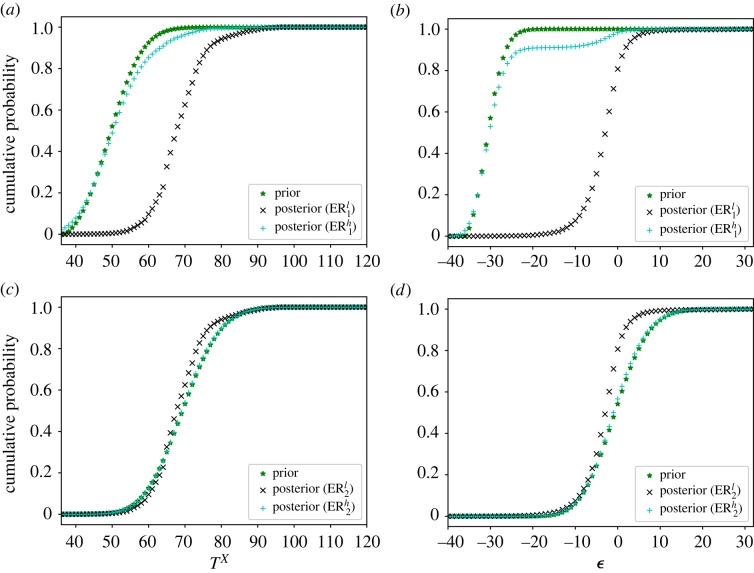
Fitting of the marginal distributions of *T*^*X*^ (*a,c*) and ϵ (*b,d*) in the Canadian dataset. The upper row contains the ER_1_ scenarios and the lower row the ER_2_ scenarios.

A quantitative comparison can be seen in table 5, which contains the mean values, variances, correlation and probability of truncation computed with each model. The results confirm the findings from figure 7. The quantities that are most affected due to the choice of a particular model are the variance of *Y* and the correlation between *X* and *Y*. The reason why variable *Y* appears to be more sensitive to each model, compared to *X*, may be the lack of information in the dataset. There are only 448 uncensored female lifetimes in the dataset, as compared to 1242 uncensored male observations. With respect to the dependence between *X* and *Y*, all models that are not strongly influenced by the choice of a prior distribution present levels of correlation around 0.5. This is in agreement with previous studies on this dataset. In particular, in comparison with the results of Arias & Cirillo [20], correlation does not appear to be highly affected by left-truncation. On the other hand, the expectation of *X* is around four years smaller if we take truncation into account, while the expectation of *Y* is reduced by 2 years. The variances are also significantly larger if we account for left-truncation, as it gives a larger weight to the left tail of the distribution. If we look at the probability of truncation, both the ${\mathrm{ER}}_{1}^{l}$ and ${\mathrm{ER}}_{2}^{l}$ estimators give a probability of almost 20%. Although this is notably smaller than the probability of truncation considered in §5.1, it is large enough to be non-negligible, in light of the comparison with the results of Arias & Cirillo [20]. The distributions of the truncation variables can be seen in figure 8, showing that the stationary assumption is not verified in this particular dataset.

**Table 5.** Table with the mean values and variances of *X* and *Y*, the correlation between *X* and *Y* and the probability of truncation using the Canadian dataset.

	mean(*X*)	mean(*Y*)	var(*X*)	var(*Y*)	corr(*X*, *Y*)	p(A)
prior_1_	53.8337	50.7572	8.1668	15.3958	0.1753	0.2919
prior_2_	79.0723	83.4871	69.1529	62.5827	0.4235	0.2668
${\mathrm{ER}}_{1}^{l}$	81.5889	84.8201	113.6054	93.0486	0.5233	0.1840
${\mathrm{ER}}_{1}^{h}$	72.9873	72.3154	204.6247	244.5786	0.9128	0.1161
${\mathrm{ER}}_{2}^{l}$	80.6768	84.4110	117.8567	81.6205	0.3706	0.2070
${\mathrm{ER}}_{2}^{h}$	79.2383	83.5579	73.0210	64.1661	0.4194	0.2594
copula	80.2418	83.3962	109.3485	83.5461	0.5145	—
KM	81.5813	87.0393	124.3929	100.5714	—	—

**Figure 9.**
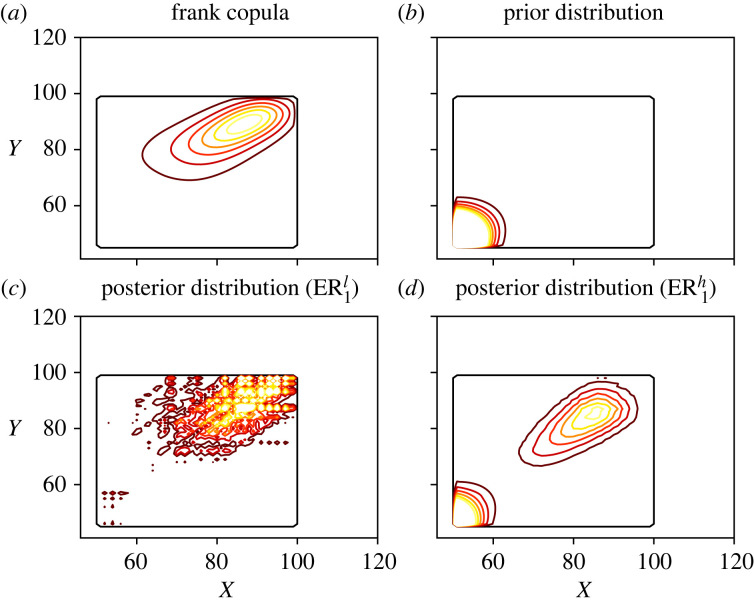
Contour plots of the reference, prior and posterior distributions using the Canadian dataset, under scenario ER_1_. The levels for each curve are the same in all plots. On the upper row the copula and prior distributions are presented, while the lower row contains the ER_1_ posterior distributions with low (*a,c*) and high (*b,d*) strengths of belief.

**Figure 10.**
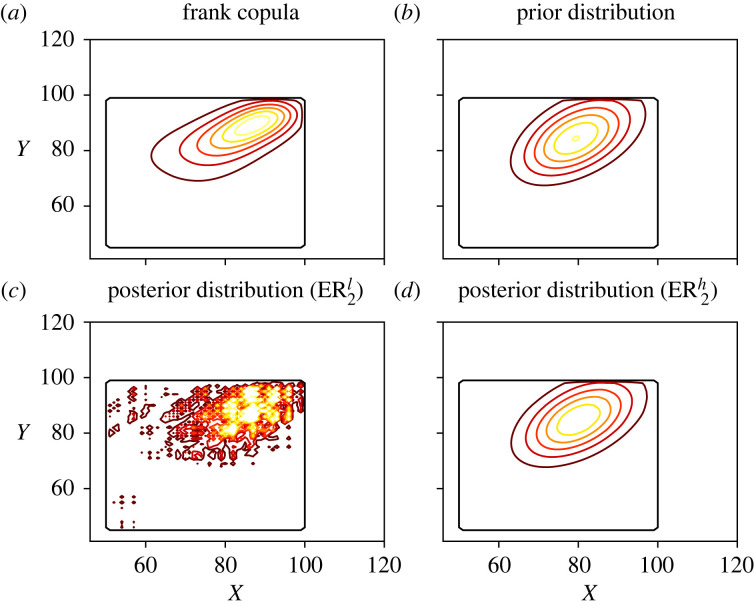
Contour plots of the reference, prior and posterior distributions using the Canadian dataset, under scenario ER_2_. The levels for each curve are the same in all plots. On the upper row the copula and prior distributions are presented, while the lower row contains the ER_2_ posterior distributions with low (*a,c*) and high (*b,d*) strengths of belief.

Finally, in figures 9 and 10, the distributions obtained with the ER and copula methods are shown. The prior distributions are also shown for comparison. The conclusions are similar to those of the previous experiments: the ER^*l*^ estimates are very similar in both scenarios and barely affected by the choice of a prior distribution. On the other hand, the ER^*h*^ estimates are highly influenced by the prior distribution. In fact, since the second prior is based on the observations, the ${\mathrm{ER}}_{2}^{h}$ estimate shows hardly any difference with respect to this prior. Thus, the contrast between the ${\mathrm{ER}}_{1}^{h}$ and ${\mathrm{ER}}_{2}^{h}$ estimates illustrates how different prior distributions can be used to introduce different features in the posterior distribution. Compared to the copula model, the ${\mathrm{ER}}_{1}^{l}$ and ${\mathrm{ER}}_{2}^{l}$ estimates present some non-negligible contours in areas with a large age difference, due to their presence in the dataset. These observations may correspond to parent-child couples instead of co-living partners, which would be relevant to maintain in the distribution if these observations are of interest. Otherwise, smoothing techniques can be applied to reduce the effect of overfitting.

## 6. Conclusion

We have proposed a novel estimation approach for bivariate LTRC observations using RUPs and the EM algorithm. The algorithm returns not only the distribution of the observed pair (*X*, *Y*), but also the distribution of the truncation variables. This can be of interest to analyse the mechanisms that generate biases in the observations, as well as to check whether the stationary condition can be applied. Analogously to the reference B-RUP model of Bulla *et al.* [2], the proposed ER algorithm benefits from the inclusion of experts’ knowledge in the form of a prior distribution, following the Bayesian paradigm.

Performances have been tested using simulated and empirical LTRC data, showing that the algorithms are able to recover the reference distribution even under substantial amounts of left-truncation and right-censoring. The proposed methodology has also been compared to the Frank copula employed in [21], producing similar results, and thus confirming the reliability of the new approach. Lastly, the effect of left-truncation has been analysed by comparing with the results obtained in [20], which only takes right-censoring into account.

Future lines of work involve extending the one-factor model of Bulla *et al.* [2], to cope with multivariate situations. This extension is straightforward in the absence of censoring and truncation, but it is more involved in a realistic and general setting. Another relevant research direction should aim at generalizing the B-RUP to deal with other forms of dependence, in particular including negative correlation and nonlinear effects.

## Data Availability

This article has no additional data.

## Footnotes

1 This condition is satisfied, for example, in lifetime follow-up studies [6], where *T* is the age at which an individual joins the study and *C* is the age at which they drop out.

2 By equation (3.6) the strength of belief is already implicit in *ω* and *β*.
